# Early Levosimendan Administration Improved Weaning Success Rate in Extracorporeal Membrane Oxygenation in Patients With Cardiogenic Shock

**DOI:** 10.3389/fcvm.2022.912321

**Published:** 2022-06-30

**Authors:** Yu-Wen Chen, Wei-Chieh Lee, Po-Jui Wu, Hsiu-Yu Fang, Yen-Nan Fang, Huang-Chung Chen, Meng-Shen Tong, Pei-Hsun Sung, Chieh-Ho Lee, Wen-Jung Chung

**Affiliations:** ^1^Division of Thoracic and Cardiovascular Surgery, Department of Surgery, Kaohsiung Chang Gung Memorial Hospital and Chang Gung University College of Medicine, Kaohsiung, Taiwan; ^2^College of Medicine, Institute of Clinical Medicine, National Cheng Kung University, Tainan, Taiwan; ^3^Division of Cardiovascular Medicine, Chi-Mei Medical Center, Tainan, Taiwan; ^4^Division of Cardiology, Department of Internal Medicine, Kaohsiung Chang Gung Memorial Hospital, Chang Gung University College of Medicine, Kaohsiung, Taiwan; ^5^Division of Cardiology, Department of Internal Medicine, Fangliao General Hospital, Pingtung, Taiwan

**Keywords:** acute decompensated heart failure (ADHF), cardiogenic shock, venoarterial extracorporeal membrane oxygenation, levosimendan, weaning

## Abstract

**Background:**

Venoarterial extracorporeal membrane oxygenation (VA-ECMO) has been increasingly used in patients with refractory cardiogenic shock (CS) or out-of-hospital cardiac arrest. It is difficult to perform VA-ECMO weaning, which may cause circulatory failure and death. Levosimendan is an effective inotropic agent used to maintain cardiac output, has a long-lasting effect, and may have the potential benefit for VA-ECMO weaning. The study aimed to explore the relationship between the early use of levosimendan and the rate of VA-ECMO weaning failure in patients on VA-ECMO support for circulatory failure.

**Methods:**

All patients who underwent VA-ECMO in our hospital for CS between January 2017 and December 2020 were recruited in this cohort study and divided into two groups: without and with levosimendan use. Levosimendan was used as an add-on to other inotropic agents as early as possible after VA-ECMO setting. The primary endpoint was VA-ECMO weaning success, which was defined as survival without events for 24 h after VA-ECMO withdrawl. The secondary outcomes were cardiovascular and all-cause mortality at the 30-day and 180-day follow-up periods post-VA-ECMO initialization.

**Results:**

A total of 159 patients were recruited for our study; 113 patients were enrolled in the without levosimendan-use group and 46 patients were enrolled in the levosimendan-use group. In levosimendan-use group, the patients received levosimendan infusion within 24 h after VA-ECMO initialization. Similar hemodynamic parameters were noted between the two groups. Poorer left ventricular ejection fraction and a higher prevalence of intra-aortic balloon pumping were observed in the levosimendan group. An improved weaning rate (without vs. with: 48.7 vs. 82.6%; *p* < 0.001), lower in-hospital mortality rate (without vs. with: 68.1 vs. 43.5%; *p* = 0.007), and 180-day cardiovascular mortality (without vs. with: 75.3 vs. 43.2%; *p* < 0.001) were also noted. Patients administered with levosimendan also presented a lower rate of 30-day (without vs. with: 75.3 vs. 41.3%; *p* = 0.034) and 180-day (without vs. with: 77.0 vs. 43.2%; *p* < 0.001) all-cause mortality.

**Conclusion:**

Early levosimendan administration may contribute to increasing the success rate of VA-ECMO weaning and may help to decrease CV and all-cause mortality.

## Background

Despite improvements in critical care medicine, the mortality and morbidity of cardiogenic shock (CS) are still high (1). CS is a high-acuity, potentially complex, and hemodynamically diverse state of end-organ hypoperfusion and causes multiple organ failures. Acute coronary syndrome with left ventricular (LV) dysfunction or acute myocarditis with severe acute decompensated heart failure (ADHF), malignant arrhythmias, or severe valvular disease is a possible reason for CS (2). The evidence for the use of vasoactive agents in CS is uncertain (3). The use of venoarterial extracorporeal membrane oxygenation (VA-ECMO) may help physicians to deal with patients with CS (4, 5). However, studies evaluating outcomes after VA-ECMO initiation have reported a wide difference in the in-hospital mortality, ranging from 10 to 90% according to the etiologies of CS, and the worst outcome was cardiac arrest associated CS (6, 7). The use of VA-ECMO is associated with an increased risk of adverse effects such as exacerbated systemic inflammatory response syndrome, catheter-related infection and thrombosis, acute kidney injury, and new organ dysfunction, especially during long-term use (8–10).

Levosimendan is a calcium-sensitizing inotropic agent with cardioprotective effects. It provides systemic, coronary, and pulmonary vasodilatory properties, and has been approved for the treatment of ADHF (11, 12). However, information on levosimendan administration for CS or VA-ECMO weaning is still questionable. Due to the vasodilatory properties of levosimendan, its initiation may worsen the status of shock. Therefore, levosimendan administration is recommended in combination with other vasoactive agents for systolic blood pressure (SBP) ≤ 90 mmHg or alone for SBP greater than 90 mmHg (13, 14). For patients with refractory CS, a combination of dobutamine and levosimendan provided better survival rates (15). For patients with severe LV dysfunction (ejection fraction < 30%), a combination of vasoactive agents and levosimendan could improve cardiac index and reduce systolic vascular resistance (16). The theoretically specific features of levosimendan for critical CS are as follows: (1) inotropic effect with respect to myocardial oxygen balance; (2) lack of proarrhythmic effect or interaction with β-blockers; (3) systemic, pulmonary, renal, and coronary vasodilation; and (4) cardioprotective effects against ischemia/reperfusion injury (11, 17, 18). In addition, the long-lasting action of active metabolites of levosimendan provides continuous support during the critical immediate post-VA-ECMO period and provides opportunities for medical modification (19). Even with the advancement of critical care medicine for VA-ECMO care, the morbidity and mortality remain high, ranging between 60 and 70%, and the rate of weaning failure was still high (20–22). The use of levosimendan may provide a potential benefit in terms of VA-ECMO weaning success, reduce the duration of mechanical support, and minimize severe complications (23, 24). Two studies concluded that levosimendan use in CS might reduce all-cause mortality and facilitate successful weaning from VA-ECMO (25, 26). However, two recent studies showed that levosimendan did not improve the rate of successful VA-ECMO weaning in patients with refractory CS (27, 28). However, no large, randomized studies have been performed to prove the impact of the combination of levosimendan in CS patients with VA-ECMO support, and previous non-randomized studies have not provided consistent results.

Therefore, this study aimed to evaluate the impact of early administration of levosimendan on VA-ECMO weaning success and associated outcomes in patients with CS under VA-ECMO support.

## Methods

### Patient Population

Between January 2017 and December 2020, 159 CS patients received VA-ECMO support and did not require cardiac surgery. We excluded patients who experienced immediate death and poor neurologic response after VA-ECMO initialization. All the 159 patients could maintain a hemodynamic condition with mechanical support devices and/or the use of combined high-dose vasoactive agents, and they were admitted to the cardiac care unit for VA-ECMO care. A total of 113 patients were enrolled in the without levosimendan-use group and 46 patients were enrolled in the levosimendan-use group. In our VA-ECMO cohort study, levosimendan infusion was administrated to the patients as soon as possible if the physicians decided to use it after the VA-ECMO setting. In the levosimendan-use group, the patients received levosimendan infusion within 24 h after VA-ECMO initialization and the median time was 8 h. Data on comorbidities, hemodynamic condition, echocardiographic and laboratory findings, use of mechanical devices, consumption of other inotropic or vasopressor agents, frequency of hospitalization for heart failure (HF), cardiovascular (CV), and all-cause mortality were compared between the two groups.

### Ethics Statement

This retrospective study conforms to the ethical guidelines of the 1975 Declaration of Helsinki. The requirement for informed consent was waived owing to the retrospective nature of the study. The study was approved by the institutional review committee of our institution for human research (number: 202200363B0).

### Echocardiography

Echocardiographic parameters, including LV ejection fraction (LVEF), LV end-diastolic volume, and LV end-systolic volume, were measured using a Philips IE33 ultrasound system. They were quantified using the two-dimensional guided biplane Simpson’s method of disc measurement by echocardiography. Echocardiography was performed in the VA-ECMO setting.

### Venoarterial Extracorporeal Membrane Oxygenation Weaning Protocol

In consideration of the duration required for recovery of the stunned myocardium after CS, all patients received VA-ECMO support for at least 48 h before weaning was attempted. If the patient’s hemodynamic conditions stabilized and echocardiographic parameters revealed improving LVEF (increased ≥ 25% when compared with baseline LVEF), the inotropic agent was tapered gradually. The mixed venous oxygen saturation was ≥ 70% without any deterioration in hemodynamic status. Pump flow reduced gradually to < 1 l/min. Finally, VA-ECMO was withdrawn when the patient’s hemodynamic status was stable. If the patients did not meet the criteria of weaning protocol, we did not withdraw the VA-ECMO support and may refer them for heart transplantation or a ventricular assistance device.

### Levosimendan Infusion

Levosimendan infusion started at 0.05 μg/kg/min for 24 h if SBP did not decrease by ≥ 10% or was less than 90 mmHg. If SBP decreases by ≥ 10% or is less than 90 mmHg after levosimendan infusion, levosimendan administration will be put on hold for 2 h and fluid resuscitation will be performed, or the dosage of other vasoactive agents will be increased. Thereafter, 24 h later, levosimendan infusion will be titrated to 0.1–0.2 μg/kg/min gradually until the 48-h administration is complete. All patients received levosimendan for at least 48 h.

### Definition

Cardiogenic shock was defined as SBP ≤ 90 mmHg for ≥ 30 min or the use of pharmacological and/or mechanical support to maintain an SBP ≥ 90 mmHg (2). Hospitalization for HF was defined as the occurrence of HF events falling within classes II–IV of the New York Heart Association Functional Classification in the absence of other alternative diagnoses. CV mortality was defined as sudden death related to arrhythmia, HF, or myocardial infarction. All-cause mortality was defined as death related to any cause, including sudden death due to undefined reasons such as natural disease course, sepsis, malignancy, and cardiovascular death. Weaning success was defined as survival without events for 24 h after VA-ECMO withdrawl.

### Study Endpoint

The study endpoints were weaning success, in-hospital mortality, CV mortality, and all-cause mortality at 30-day and 180-day follow-up periods post-VA-ECMO initialization. The study endpoints were reviewed by VA-ECMO team to adjust the outcomes.

### Statistical Analysis

Numerical data are presented as the mean ± standard deviation or the number of patients (percentages) for normally distributed variables. The characteristics of the study groups were compared using the *t*-test for continuous variables and Chi-square test for categorical variables. Univariate and multivariate logistic regression analyses for weaning success were performed to determine significant determinants. The factors of significant difference in the odds ratio (OR) for weaning success in univariate logistic regression analyses were included for multivariate logistic regression analysis. Kaplan–Meier curves were created to illustrate the 30-day and 180-day all-cause mortality rates for the two groups. Statistical analyses were performed using statistical software (SPSS Statistics for Windows version 22, IBM. Corp., Armonk, NY, United States) and a two-sided *p* < 0.05 was defined as statistically significant.

## Results

### Baseline Characteristics

The baseline characteristics of the study population are shown in Table 1. Between the groups without and with levosimendan use, the mean age and prevalence of males did differ significantly. The indications for VA-ECMO, hemodynamic condition, and comorbidities did not differ between the two groups. A higher level of serum lactic acid [without vs. with: 83.7 (73.0–105.0) mmol/L vs. 58.4 (40.6–69.3) mmol/L; *p* = 0.001] and lower serum albumin (without vs. with: 2.6 ± 0.7 g/dL vs. 3.0 ± 0.7 g/dL; *p* = 0.001) were noted in the without levosimendan-use group. A higher level of serum lactic acid [without vs. with: 18.4 (15.9–23.1) mmol/L vs. 58.4 (40.6–69.3) mmol/L; *p* = 0.011] was still noted 24 h later in the without levosimendan-use group. A non-significant trend of high 24-h lactic acid clearance [without vs. with: 61.5 (57.1–71.8)% vs. 60.9 (53.0–72.3)%; *p* = 0.070] was noted in the levosimendan-use group. Poorer LVEF was noted in the levosimendan-use group (without vs. with: 35.2 ± 17.2% vs. 30.1 ± 12.2%; *p* = 0.045). A higher prevalence of intra-aortic balloon pumping (IABP) was noted in the levosimendan-use group (without vs. with: 66.4 vs. 95.7%; *p* < 0.001). A longer duration of VA-ECMO support was shown in the levosimendan-use group [without vs. with: 4.7 (3.8–5.9) days vs. 7.1 (5.0–7.9) days; *p* = 0.003]. In the levosimendan-use group, levosimendan infusion was temporarily halted because of a decrease in SBP in 32.6% of patients, was used with a low-dose strategy in 19.5% of patients, and arrhythmia was noted in 6.5% of patients. The median duration from levosimendan administration to VA-ECMO weaning was 4 days. During the 30-day duration, only 2 patients converted to a ventricular assistance device in the levosimendan-use group and did not differ between the two groups. During the follow-up period, only 5 patients received heart transplantation and did not differ between the two groups.

**TABLE 1:** Demographics and clinical characteristics.

Variables	Without levosimendan (*N* = 113)	With levosimendan (*N* = 46)	*P*-value
**Demographic**			
Age (years)	59 ± 11.4	58 ± 16.5	0.591
Male gender (%)	76 (67.3)	37 (80.4)	0.123
**Indication of VA-ECMO**			0.103
Myocardial infarction (%)	24 (21.2)	11 (23.9)	
Severe HF (%)	15 (13.3)	12 (26.1)	
Cardiac arrest (%)	74 (65.5)	23 (50.0)	
Out-of-hospital	61 (82.4)	20 (87.0)	0.755
In-hospital	13 (17.6)	3 (13.0)	
**Hemodynamic condition**			
SBP (mmHg)	88.6 ± 30.7	89.2 ± 25.0	0.956
DBP (mmHg)	55.1 ± 19.2	54.1 ± 19.2	0.842
HR (beats/min)	99.6 ± 25.1	108.4 ± 25.4	0.195
**Medical history**			
Diabetes mellitus (%)	41 (36.3)	12 (26.1)	0.267
Hypertension (%)	56 (49.6)	19 (41.3)	0.384
Prior history of stroke (%)	8 (7.1)	5 (10.9)	0.524
Coronary artery disease (%)	85 (75.2)	33 (71.7)	0.691
Prior history of heart failure (%)	17 (15.0)	3 (6.5)	0.190
End-stage renal disease	11 (9.7)	2 (4.3)	0.350
Chronic kidney disease, stage ≥3 (%)	66 (58.4)	22 (47.8)	0.291
**Laboratory data**			
** Before VA-ECMO implantation**			
BUN (mg/dl)	21.0 (18.0–23.0)	20.0 (17.0–25.0)	0.389
Creatinine (mg/dL)	1.6 (1.5–1.8)	1.4 (1.3–1.7)	0.738
BNP (pg/ml)	761.0 (485.2–1118.8)	742.5 (394.0–1372.9)	0.499
Troponin-I (ng/mL)	8.5 ± 3.0	9.1 ± 2.1	0.840
Lactic acid (*mmol*/*L)*	83.7 (73.0–105.0)	58.4 (40.6–69.3)	0.001
Albumin (g/dl)	2.6 ± 0.7	3.0 ± 0.7	0.001
** 24 h later**			
BUN (mg/dl)	26.0 (21.9–33.0)	23.0 (19.2–36.0)	0.439
Creatinine (mg/dL)	1.8 (1.2–2.5)	1.2 (1.0–1.9)	0.125
Lactic acid (*mmol*/*L)*	18.4 (15.9–23.1)	15.4 (12.7–22.1)	0.011
Albumin (g/dl)	2.8 ± 0.5	2.8 ± 0.4	0.430
** 24-H clearance**			
The change of BUN (%)	16.4 (0–36.4)	25.4 (–15.4–64.1)	0.583
The change of creatinine (%)	–1.6 (–16.4–21.7)	–15.5 (–28.0–22.5)	0.354
Lactic acid clearance (%)	61.5 (57.1–71.8)	60.9 (53.0–72.3)	0.070
**Echocardiographic parameters**			
LVEF (%)	35.2 ± 17.2	30.1 ± 12.2	0.045
LVEDV (ml)	130.0 (115.9–147.8)	125.0 (101.1–146.1)	0.353
LVESV (ml)	83.0 (62.0–106.2)	92.0 (68.8–102.4)	0.721
LAD (mm)	52.5 ± 14.3	51.6 ± 9.9	0.732
The grade of AR ≥ 3 (%)	6 (5.3)	1 (2.2)	0.674
The grade of MR ≥ 3 (%)	12 (10.6)	5 (10.9)	1.000
The grade of TR ≥ 3 (%)	7 (6.2)	2 (4.3)	1.000
TRPG (mmHg)	23.1 ± 13.5	21.9 ± 8.5	0.689
**Mechanical support**			
IABP (%)	79 (66.4)	44 (95.7)	< 0.001
Ventilator (%)	110 (97.3)	43 (93.5)	0.357
**Inotropic or vasopressor agents**			
Epinephrine (%)	37 (32.7)	10 (21.7)	0.185
Norepinephrine (%)	69 (61.1)	26 (56.5)	0.598
Dopamine (%)	71 (62.8)	25 (54.3)	0.373
Dobutamine (%)	12 (10.6)	7 (15.2)	0.427
Milrinone (%)	7 (6.2)	5 (10.9)	0.331
VA-ECMO duration (days)	4.7 (3.8–5.9)	7.1 (5.0–7.9)	0.003
The use of distal protection (%)	8 (7.1)	8 (17.4)	0.077
Ventricular assistance device (%)	0	2 (4.3)	0.082
Heart transplantation (%)	2 (1.8)	3 (6.5)	0.146

*Data are expressed as mean ± standard deviation or as a number (percentage) or medians with interquartile ranges if non-normally distributed variables. AR, aortic regurgitation; BNP, brain natriuretic peptide; BUN, blood urea nitrogen; CrCl, creatinine clearance; DBP, diastolic blood pressure; VA-ECMO, venoarterial extracorporeal membrane oxygenation; HF, heart failure; HR, heart rate; IABP, intra-aortic balloon pumping; LVEF, left ventricular ejection fraction; LVEDV, left ventricular end-diastolic volume; LVESV, left ventricular end-systolic volume; LAD, left atrial dimension; MR, mitral regurgitation; SBP, systolic blood pressure; TR, tricuspid regurgitation; TRPG, tricuspid regurgitation pressure gradient.*

### The Rate of Weaning Success and In-Hospital Mortality and Short-Term Outcomes

A significantly higher rate of VA-ECMO weaning success was noted in the levosimendan-use group (without vs. with: 48.7 vs. 82.6%; *p* < 0.001) (Table 2). A lower incidence of in-hospital mortality was noted in the levosimendan-use group (without vs. with: 68.1 vs. 43.5%; *p* = 0.007). During the 30-day follow-up period, a lower incidence of all-cause mortality was noted in the levosimendan-use group (without vs. with: 60.7 vs. 41.3%; *p* = 0.034). During the 180-day follow-up period, a lower incidence of CV mortality (without vs. with: 75.3 vs. 43.2%; *p* < 0.001) and all-cause mortality (without vs. with: 77.0 vs. 43.2%; *p* < 0.001) were noted in the levosimendan-use group.

**TABLE 2:** In-hospital and short-term outcomes.

Variables	Without levosimendan (*N* = 113)	With levosimendan (*N* = 46)	*P*-value
Weaning success (%)	55 (48.7)	38 (82.6)	< 0.001
In-hospital mortality (%)	77 (68.1)	20 (43.5)	0.007
**Heart-failure hospitalization**			
180-day (%)	23 (16.1)	3 (7.3)	0.172
**Cardiovascular mortality**			
30-day (%)	65 (59.6)	19 (41.3)	0.052
180-day (%)	70 (75.3)	19 (43.2)	< 0.001
**All-cause mortality**			
30-day (%)	68 (60.7)	19 (41.3)	0.034
180-day (%)	77 (77.0)	19 (43.2)	< 0.001

*Data are expressed as a number (percentage).*

### Kaplan–Meier Curves of All-Cause Mortality Between the Two Groups

**FIGURE 1:**
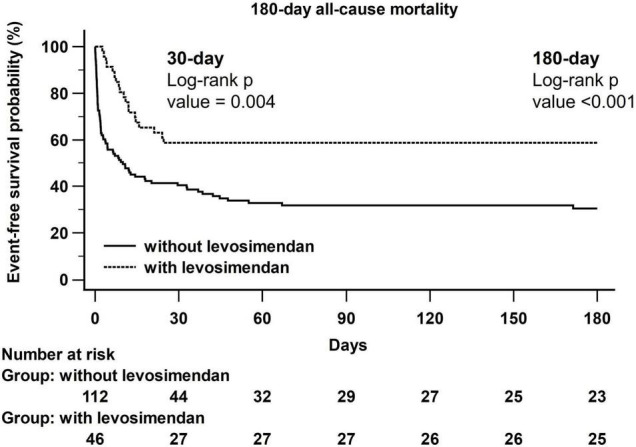
Kaplan–Meier curves of all-cause mortality between without and with levosimendan-use groups.

Figure 1 shows a Kaplan–Meier curve illustrating the difference in all-cause mortality at 30 days (log-rank *p* = 0.004) and 180 days (log-rank *p* < 0.001) between the without levosimendan-use group and levosimendan-use group.

### Univariate and Multivariate Logistic Regression Analyses of Predictors of Venoarterial Extracorporeal Membrane Oxygenation Weaning Success

Univariate logistic regression analyses showed that older age, renal insufficiency (CrCl ≤ 60 mL/min), IABP use, levosimendan administration, and a lower serum lactic acid level were significant predictors of successful VA-ECMO weaning (Table 3). When multivariate logistic regression analyses were performed, older age [OR: 0.958, 95% confidence interval (CI): 0.923–0.995; *p* = 0.026], levosimendan administration (OR: 3.072, 95% CI: 1.019–9.263; *p* = 0.046), and a lower serum lactic acid level (OR: 0.980, 95 CI: 0.972–0.989; *p* < 0.001) were significant predictors of VA-ECMO weaning success.

**TABLE 3:** Univariate and multivariate logistic regression analyses of predictors of VA-ECMO weaning success.

	Univariate analysis			Multivariate analysis		
Variables	OR	95% CI	*p*-value	OR	95% CI	*p*-value
Cardiac arrest	0.516	0.259–1.027	0.060			
Age	0.968	0.942–0.995	0.020	0.958	0.923–0.995	0.026
Female	0.749	0.371–1.513	0.421			
Diabetes mellitus	1.042	0.524–2.069	0.907			
Prior HF history	0.665	0.258–1.716	0.399			
Chronic kidney disease, stage ≥3	0.408	0.207–0.804	0.010	0.471	0.198–1.122	0.089
ESRD	1.321	0.388–4.495	0.656			
Coronary artery disease	1.330	0.643–2.754	0.442			
IABP use	2.818	1.350–5.880	0.006	2.497	0.988–6.313	0.053
Levosimendan administration	5.683	2.232–14.467	< 0.001	3.072	1.019–9.263	0.046
Double vasoactive agents	0.682	0.349–1.331	0.262			
Distal perfusion	0.405	0.142–1.154	0.091			
LVEF (%)	0.993	0.971–1.017	0.574			
LVEDV (ml)	0.999	0.995–1.004	0.801			
LVESV (ml)	0.999	0.993–1.004	0.660			
LAD (mm)	0.998	0.970–1.027	0.900			
MR grade ≥ 3	0.611	0.222–1.683	0.341			
TR grade ≥ 3	0.437	0.113–1.698	0.232			
TRPG (mmHg)	0.962	0.922–1.003	0.072			
BUN (mg/dl)	0.989	0.975–1.004	0.137			
Troponin-I (ng/mL)	1.008	0.978–1.039	0.589			
Lactic acid (*mmol*/*L)*	0.977	0.968–0.985	< 0.001	0.980	0.972–0.989	< 0.001
BUN clearance	0.654	0.390–1.095	0.106			
Lactic acid clearance	0.625	0.337–1.159	0.135			
Recent two-year period	0.929	0.481–1.793	0.826			

*BUN, blood urea nitrogen; CI, confidence interval; VA-ECMO, venoarterial extracorporeal membrane oxygenation; ESRD, end-stage renal disease; HF, heart failure; IABP, intra-aortic balloon pumping; LVEF, left ventricular ejection fraction; LVEDV, left ventricular end-diastolic volume; LVESV, left ventricular end-systolic volume; LAD, left atrial dimension; MR, mitral regurgitation; OR, odd ratio; TR, tricuspid regurgitation; TRPG, tricuspid regurgitation pressure gradient.*

### The 30-Day and 180-Day All-Cause Mortality Rate in the Subgroups With Cardiac Arrest, Non-cardiac Arrest, and Chronic Kidney Disease, Stage ≥3

**FIGURE 2:**
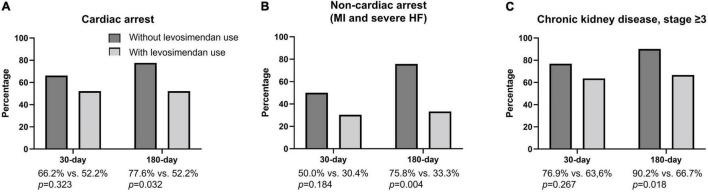
30-day and 180-day all-cause mortality rates in subgroups with cardiac arrest, non-cardiac arrest, and chronic kidney disease, stage ≥3. **(A)** In patients experiencing cardiac arrest, the incidence of all-cause mortality did not differ between the two groups at the 30-day follow-up period (without vs. with: 66.2 vs. 52.2%; *p* = 0.323) but showed a significant difference at the 180-day follow-up period (without vs. with: 77.6 vs. 52.2% *p* = 0.032) when the without levosimendan use was compared to the levosimendan-use group. **(B)** In patients experiencing non-cardiac arrest (MI and severe HF), the incidence of all-cause mortality did not differ between two groups at the 30-day follow-up period (without vs. with: 50.0 vs. 30.4%; *p* = 0.184) but showed a significant difference at the 180-day follow-up period (without vs. with: 75.8 vs. 33.3% *p* = 0.004) when the without levosimendan use was compared to the levosimendan-use group. **(C)** In patients with chronic kidney disease, stage ≥3, the incidence of all-cause mortality did not differ between the two groups at the 30-day follow-up period (without vs. with: 76.9 vs. 63.6%; *p* = 0.267) but showed a significant difference at the 180-day follow-up period (without vs. with: 90.2 vs. 66.7%: *p* = 0.018) when the without levosimendan use was compared to the levosimendan-use group.

In patients experiencing cardiac arrest and non-cardiac arrest (Figures 2A,B), a significantly higher incidence of all-cause mortality was noted in the levosimendan-use group at the 180-day follow-up period (cardiac arrest; without vs. with: 77.6 vs. 52.2% *p* = 0.032; non-cardiac arrest; without vs. with: 75.8 vs. 33.3% *p* = 0.004). In patients with chronic kidney disease, stage ≥3 (Figure 2C), a significantly higher incidence of all-cause mortality was noted in the levosimendan-use group at the 180-day follow-up period (without vs. with: 90.2 vs. 66.7% *p* = 0.018).

## Discussion

In the present study of CS patients with VA-ECMO support, early levosimendan administration provided a higher rate of VA-ECMO weaning success, and patients with levosimendan administration presented better in-hospital, 30-day, and 180-day all-cause mortality. Other predictors of VA-ECMO weaning success were younger age and lower serum lactic acid levels. The use of IABP and double vasoactive agents and the enrolled period did not achieve significance. In the subgroups with cardiac arrest and renal insufficiency chronic kidney disease, stage ≥3, a lower prevalence of 180-day all-cause mortality was noted in patients using levosimendan.

### Levosimendan Administration for Weaning Venoarterial Extracorporeal Membrane Oxygenation

According to previous studies, levosimendan administration significantly increased successful weaning rates in patients with cardiopulmonary support, especially in those with combined poor LV performance (23–26). Regarding the issue of VA-ECMO for patients with CS, the enrolled studies were non-randomized studies with a limited number of patients (25). However, a recent study using propensity score matching showed that levosimendan did not improve the rate of successful VA-ECMO weaning in patients with refractory CS (27). On the other hand, one recent meta-analysis included the new report and still confirms that levosimendan may be an effective option to facilitate weaning from VA-ECMO and reduce mortality risk, but the conclusion must be interpreted with caution because of the potential limitations of the currently available studies (29). However, Guilherme stated that 42 patients who received VA-ECMO for less than 48 h were excluded, as were patients with refractory cardiac arrest (27). In previous studies, most studies combined patients with cardiac surgery (27, 30). In our study, we did not exclude patients with cardiac arrest despite high mortality and did not enroll patients with post-cardiac surgery status. Our levosimendan administration strategy was implemented as early as possible after VA-ECMO initiation. Other vasoactive agents may also cause vasoconstriction and worsen perfusion. Therefore, it is reasonable to use a combination of levosimendan as early as possible to reduce vasoconstriction and increase organ perfusion. We compared the effect of levosimendan add-on with other vasoactive agents and early administration of levosimendan in patients with CS on VA-ECMO support. In our study, patients experiencing cardiac arrest also presented a better 180-day prognosis for all-cause mortality.

### Levosimendan Administration for Cardiorenal Syndrome

Levosimendan can cause renal arterial and venous dilatation and may also reverse cardiorenal syndrome in critical conditions (30, 31). One study reported that levosimendan increases the glomerular filtration rate to a greater extent than dobutamine in patients with chronic HF and renal impairment (32). In CS, renal perfusion decreases due to low cardiac output and causes acute or chronic kidney injury. Patients who developed renal insufficiency and required replacement treatment while on VA-ECMO had higher hospital mortality (10). In our study, there was an improvement in the mean serum creatinine level and a decreased percentage when compared with the baseline level, but it did not achieve statistical significance. We still noted a trend of renal protection in the levosimendan-use group (33, 34).

### The Influence of Concomitant Intra-Aortic Balloon Pumping Counterpulsation for Weaning Venoarterial Extracorporeal Membrane Oxygenation

According to previous studies, the influence of concomitant IABP counterpulsation on the weaning success of extracorporeal life support system was still a controversial issue and presented different results in the patients with different etiologies related to CS (35–37). In our study, a higher prevalence of IABP use in the levosimendan-use group may affect the clinical outcomes and VA-ECMO weaning success even though no significant difference was noted in multivariate logistic regression analyses.

### Limitations

Although the study provides substantial evidence of better treatment outcomes following levosimendan administration in patients with CS on VA-ECMO support, there are limitations to be acknowledged. This was a retrospective study and included data from only one medical center, with the choice of levosimendan administration being solely dependent on the physician’s expertise, and thereby was a limitation. In our study, the physician in the cardiac care unit decided whether or not to use levosimendan after stabilizing the hemodynamic condition after VA-ECMO setting and/or high-dose vasoactive agents. The strength of recommendation for levosimendan infusion may be influenced by LVEF and a high need for vasopressors/inotropes at that moment. Additionally, we could not control the bias of being alive at that point in the time of levosimendan use. The confounding indications and the difficulties surrounding an appropriate definition of weaning success were the important limitations of this study. Prospective studies involving larger sample sizes or randomized studies are needed to validate our findings, especially for patients who do not require cardiac surgery and for the recovery of renal function under CS status. Currently, two ongoing randomized studies [WEANILEVO trial (NCT04158674) and LEVOECMO trial (NCT04728932)] are performed to test if the early administration of levosimendan can facilitate and accelerate VA-ECMO weaning, and translate into less morbidity, reduced length of stays in the intensive care unit and hospital. In our study, the administration of levosimendan was performed as early as possible after VA-ECMO initialization, and it seems to increase the success rate of VA-ECMO weaning and may bring survival benefits.

## Conclusion

Early levosimendan administration may contribute to increasing the success rate of VA-ECMO weaning and may help to decrease CV and all-cause mortality. Younger age, levosimendan administration, and a low initial lactic acid level were predictors of VA-ECMO weaning success. Levosimendan administration also provided a better 180-day prognosis even in patients experiencing cardiac arrest and a trend of better renal recovery under CS status.

## Data Availability Statement

The raw data supporting the conclusions of this article will be made available by the authors, without undue reservation.

## Ethics Statement

The studies involving human participants were reviewed and approved by the IRB number of this study was 202200363B0 and was approved by Ethic Committee of Kaohsiung Chang Gung Memorial Hospital. Written informed consent for participation was not required for this study in accordance with the national legislation and the institutional requirements. Written informed consent was not obtained from the individual(s) for the publication of any potentially identifiable images or data included in this article.

## Author Contributions

Y-WC and W-CL contributed to the conception, design of the study, and wrote the first draft of the manuscript. W-CL organized the database and performed the statistical analysis. P-JW, H-YF, Y-NF, H-CC, M-ST, P-HS, C-HL, and W-JC wrote sections of the manuscript. All authors contributed to manuscript revision, read, and approved the submitted version.

## Conflict of Interest

The authors declare that the research was conducted in the absence of any commercial or financial relationships that could be construed as a potential conflict of interest.

## Publisher’s Note

All claims expressed in this article are solely those of the authors and do not necessarily represent those of their affiliated organizations, or those of the publisher, the editors and the reviewers. Any product that may be evaluated in this article, or claim that may be made by its manufacturer, is not guaranteed or endorsed by the publisher.
